# Ubiquitination Is Essential for Avibirnavirus Replication by Supporting VP1 Polymerase Activity

**DOI:** 10.1128/JVI.01899-18

**Published:** 2019-01-17

**Authors:** Huansheng Wu, Liuyuan Shi, Yina Zhang, Xiran Peng, Tuyuan Zheng, Yahui Li, Boli Hu, Xiaojuan Zheng, Jiyong Zhou

**Affiliations:** a MOA Key Laboratory of Animal Virology, Department of Veterinary Medicine, Zhejiang University, Hangzhou, China; b MOE International Joint Collaborative Laboratory for Animal Health and Food Safety, Institute of Immunology and College of Veterinary Medicine, Nanjing Agricultural University, Nanjing, China; c Collaborative Innovation Center and State Key Laboratory for Diagnosis and Treatment of Infectious Diseases, First Affiliated Hospital, Zhejaing University, Hangzhou, China; University of Kentucky College of Medicine

**Keywords:** ubiquitination, VP1 protein, avibirnavirus, polymerase activity

## Abstract

Ubiquitination is critical for several cellular physical processes. However, ubiquitin modification in virus replication is poorly understood. Therefore, the present study aimed to determine the presence and effect of ubiquitination on polymerase activity of viral protein 1 (VP1) of avibirnavirus. We report that the replication of avibirnavirus is regulated by ubiquitination of its VP1 protein, the RNA-dependent RNA polymerase of infectious bursal disease virus (IBDV). *In vivo* detection revealed the ubiquitination of VP1 protein in IBDV-infected target organs and different cells but not in purified IBDV particles. Further analysis of ubiquitination confirms that VP1 is modified by K63-linked ubiquitin chain. Point mutation screening showed that the ubiquitination site of VP1 was at the K751 residue in the C terminus. The K751 ubiquitination is independent of VP1’s interaction with VP3 and eukaryotic initiation factor 4A II. Polymerase activity assays indicated that the K751 ubiquitination at the C terminus of VP1 enhanced its polymerase activity. The K751-to-R mutation of VP1 protein did not block the rescue of IBDV but decreased the replication ability of IBDV. Our data demonstrate that the ubiquitination of VP1 is crucial to regulate its polymerase activity and IBDV replication.

**IMPORTANCE** Avibirnavirus protein VP1, the RNA-dependent RNA polymerase, is responsible for IBDV genome replication, gene expression, and assembly. However, little is known about its chemical modification relating to its polymerase activity. In this study, we revealed the molecular mechanism of ubiquitin modification of VP1 via a K63-linked ubiquitin chain during infection. Lysine (K) residue 751 at the C terminus of VP1 is the target site for ubiquitin, and its ubiquitination is independent of VP1’s interaction with VP3 and eukaryotic initiation factor 4A II. The K751 ubiquitination promotes the polymerase activity of VP1 and unubiquitinated VP1 mutant IBDV significantly impairs virus replication. We conclude that VP1 is the ubiquitin-modified protein and reveal the mechanism by which VP1 promotes avibirnavirus replication.

## Abstract (precis)

Avibirnavirus protein VP1, the RNA-dependent RNA polymerase, is responsible for IBDV genome replication, gene expression, and assembly. However, little is known about its chemical modification relating to its polymerase activity. In this study, we revealed the molecular mechanism of ubiquitin modification of VP1 via a K63-linked ubiquitin chain during infection. Lysine (K) residue 751 at the C terminus of VP1 is the target site for ubiquitin, and its ubiquitination is independent of VP1’s interaction with VP3 and eukaryotic initiation factor 4A II. The K751 ubiquitination promotes the polymerase activity of VP1 and unubiquitinated VP1 mutant IBDV significantly impairs virus replication. We conclude that VP1 is the ubiquitin-modified protein and reveal the mechanism by which VP1 promotes avibirnavirus replication.

## INTRODUCTION

Ubiquitination, a highly dynamic enzyme reaction process, comprises the attachment of an ∼8-kDa ubiquitin molecule to a corresponding lysine (K) site on the target protein (1). This process involves several enzymatic reactions to transfer ubiquitin from the E1 ubiquitin activation enzyme to the E2 ubiquitin conjugation enzyme and finally to the E3 ubiquitin ligase protein, which supports ubiquitin conjugation to the target protein (2). Previous studies demonstrated that ubiquitin contained seven lysine residues that generated seven different ubiquitin chains or even multiple complex ubiquitin chains (3), and linear ubiquitin chains, a newly discovered ubiquitin chain catalyzed by the linear ubiquitin chain assemble complex (4), to regulate cellular physical process (5). The K48-linked polyubiquitin chain is commonly involved in protein degradation via the proteasome (6), whereas the K63 special ubiquitin chain is associated with protein trafficking, protein interaction, and folding maturation (7). Several studies have revealed that ubiquitination was involved in the life cycle of several viruses, e.g., influenza A virus (8–10), human immunodeficiency virus (11–13), Ebola virus (14), parainfluenza virus 5 (15), and porcine circovirus type 2 (16).

Avibirnavirus, the infectious bursal disease virus (IBDV), is a nonenveloped double-stranded RNA (dsRNA) virus, belonging to the *Birnaviridae* (17). The IBDV genome contains two segments, segment A and segment B (18, 19). IBDV genomic segment A encodes viral protein 5 (VP5), which is involved in inducing apoptosis (20–22), and the polyprotein, which is autocleaved into pVP2, VP4, and VP3 (23, 24). pVP2 is further processed into mature VP2, along with four small peptides (25, 26). Meanwhile, IBDV genomic segment B produces VP1 with an approximately molecular weight of 100 kDa, the RNA-dependent RNA polymerase (RdRp) protein of IBDV (27). VP1 is considered to form the replication complex containing genomic dsRNA and VP3 and is believed to be responsible for genome RNA transcription, replication, and VPg formation in the mature virion (28–31). Recent reports demonstrated that VP3 could promote VP1 polymerase activity *in vitro* and *in vivo* and that both VP1 and VP3 were required for translation initiation of uncapped IBDV genome dsRNA (32, 33). However, the roles of posttranslation modifications of VP1 in regulating its polymerase activity are poorly understood. Self-guanylylation of VP1 is not required for intact polymerase activity (34). To date, the relationship between ubiquitination and avibirnavirus polymerase activity is unknown. Therefore, the present study aimed to determine the presence and effect of ubiquitination on avibirnavirus VP1 polymerase activity.

We demonstrate here that VP1 is efficiently ubiquitinated at lysine residue 751 (K751), located in the C terminus of VP1 by a K63-linked ubiquitin chain. This ubiquitination was independent of VP1’s interaction with VP3 and eukaryotic initiation factor 4A II (eIF4AII). Moreover, K751 ubiquitination promotes VP1 polymerase activity and IBDV replication. We conclude that VP1 ubiquitination plays crucial roles in virus replication via controlling the polymerase activity.

## RESULTS

### Avibirnavirus polymerase protein VP1 undergoes ubiquitination during infection.

**FIG 1:**
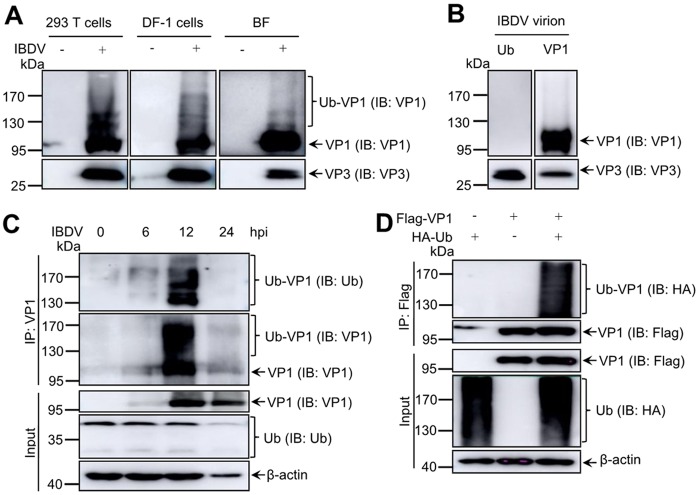
Avibirnavirus polymerase protein VP1 undergoes ubiquitination during infection. (A) Mass molecular shift modified bands of VP1 during IBDV infection. Lysates from 293T cells, DF-1 cells, and BF cells infected with IBDV were analyzed by Western blotting with mouse anti-VP1 MAb. Mock-infected cells and BF cells were used as negative controls. (B) Purified IBDV virions were probed by Western blotting with anti-VP1 MAb and anti-Ub polyclonal antibody. (C) Dynamic profiles of VP1 ubiquitination during infection. DF-1 cells infected with IBDV at 5 MOI were cultured at the indicated time points before immunoprecipitation with anti-VP1 antibody plus protein A/G. Immunoblotting was performed with anti-Ub pAb, anti-VP1 MAb, and anti-VP3 MAb. (D) VP1 was modified by ubiquitin during transfection. Flag-VP1 and HA-Ub were cotransfected into 293T cells for 48 h. Cell lysates were used for *in vivo* ubiquitination assays and subjected to Western blotting with the indicated antibodies. Flag-VP1 or HA-Ub transfected alone was used as the control.

To detect chemical modification of viral proteins during IBDV infection, ubiquitination was measured in IBDV-infected cells and target tissue using Western blotting. After 293T cells were infected with IBDV at a multiplicity of infection (MOI) of 1 for 12 h, viral protein VP1, with an approximately molecular weight of 100 to 170 kDa, could be detected using a mouse anti-VP1 monoclonal antibody (MAb) (Fig. 1A). However, VP1 proteins of this molecular weight was not exhibited in purified IBDV particles (Fig. 1B) and other viral proteins encoded by IBDV (data not shown). Consistently, this posttranslational modification was also observed in DF-1 cells and tissues obtained from chicken bursa of Fabricius (BF) after IBDV infection (Fig. 1A). To verify that the posttranslational modification of VP1 was ubiquitin modification, *in vivo* ubiquitination assays were performed during IBDV infection using anti-ubiquitin and anti-VP1 MAbs. The *in vivo* ubiquitination assays showed that VP1 protein was efficiently modified by polyubiquitin in IBDV-infected and VP1-transfected cells (Fig. 1C and D). Moreover, VP1 ubiquitination in IBDV-infected cells was gradually increased in a time-dependent manner, reaching a peak at 12 h after infection. However, the ubiquitinated VP1 decreased at 24 h postinfection. Taken together, our data clearly showed that ubiquitination of viral protein VP1 occurred only during the IBDV replication process rather than being incorporated into mature viral particles.

### VP1 protein exhibits K63-linked ubiquitination during IBDV replication.

**FIG 2:**
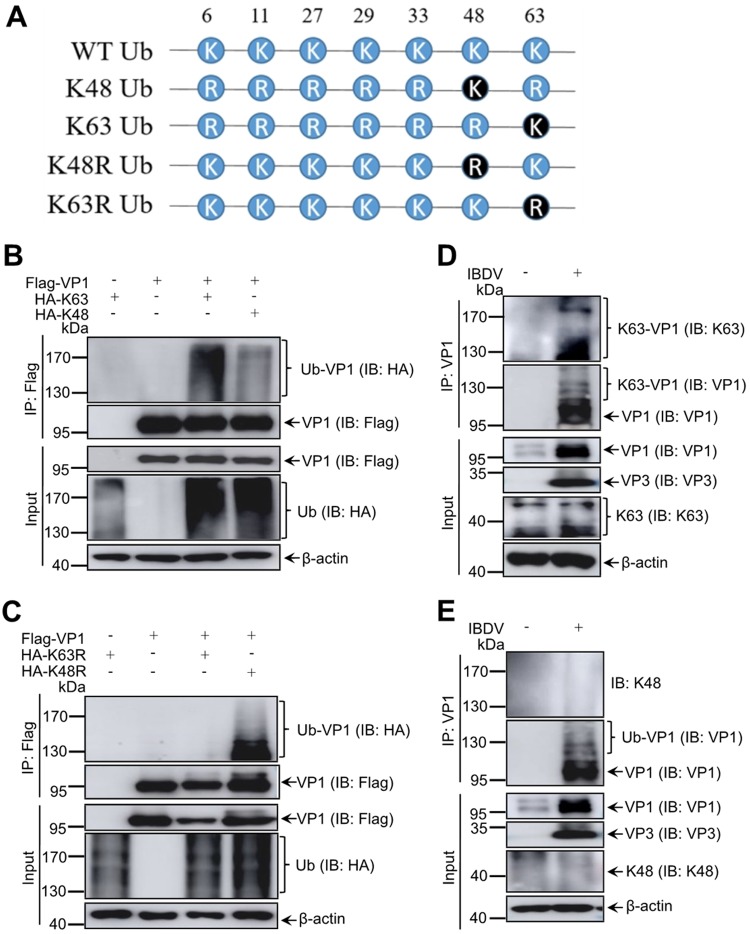
K63-linked ubiquitination of VP1 during IBDV replication. (A) Schematic diagram of ubiquitin K48 and K63 mutants. (B) VP1 was modified by K63-linked ubiquitin during transfection. 293T cells were cotransfected with Flag-VP1 and either HA-K63 or HA-K48 for 48 h. The cell lysates were subjected to *in vivo* ubiquitination assays and Western blotting with indicated antibodies. (C) VP1 was modified by K48R mutant-linked ubiquitin during transfection. 293T cells were cotransfected with Flag-VP1 and HA-K63R or HA-K48R for 48 h. The lysates were subjected to *in vivo* ubiquitination assay and Western blotting with indicated antibodies. (D and E) VP1 was modified by K63-linked ubiquitin (D), rather than by K48-linked ubiquitin (E), during virus infection. DF-1 cells infected with IBDV at 5 MOI for 12 h were used in an *in vivo* ubiquitination assay with anti-VP1 antibody and for Western blotting with K63/K48-specific antibodies. All input proteins were detected by Western blotting with the indicated antibodies; β-actin expression was used as a loading control.

Ubiquitin is composed of 72 amino acid residues, including seven lysine residues (Fig. 2A) (1). Each lysine residue could be form single or mixed polyubiquitin chain linkages, which play different roles in various cellular signaling pathway (35). To identify the specific ubiquitin chain linking to the VP1 protein, the expression plasmids of Flag-VP1 and HA-K63 (the ubiquitin mutant without K63 substitution) or HA-K48 (the ubiquitin mutant without K48 substitution) were constructed and cotransfected into 293T cells, and HA-K63 transfection alone acted as a negative control. The ubiquitination assays showed that VP1 was mainly modified by K63-linked ubiquitin compared to the K48-linked ubiquitin (Fig. 2B). HA-K63R (Lys 63 mutated to Arg) and HA-K48R (Lys 48 mutated to Arg) were used to further analyze the ubiquitin type of VP1. We observed no K63R-linked ubiquitination of VP1, while VP1 was highly efficiently ubiquitinated in the presence of K48R ubiquitin (Fig. 2C), similarly to the extent shown Fig. 2B and presumably representing K63-linked ubiquitination. Next, we detected endogenous K63-linked and K48-linked ubiquitination of VP1 during virus infection. We noted that a strong signal was observed when using an anti-K63-linked ubiquitin antibody, but a weak signal was also detected using anti-K48-linked ubiquitin antibody by Western blotting after *in vivo* ubiquitination assays with the anti-VP1 antibody during IBDV infection (Fig. 2D and E), which revealed that VP1 was mainly modified by K63-linked ubiquitin chains.

### Lys 751 of VP1 is the target site of ubiquitination.

**FIG 3:**
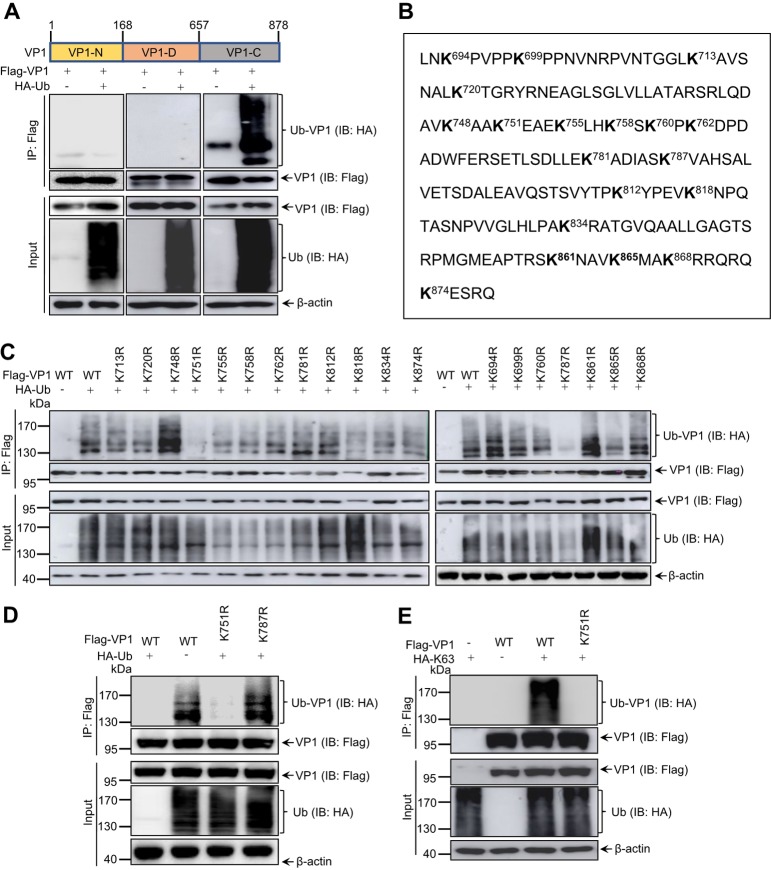
Lys 751 of VP1 is the target site for ubiquitination. (A) The C terminus of VP1 was modified by ubiquitin. 293T cells were cotransfected with HA-Ub and the indicated Flag-tag VP1 subdomains for 48 h. The lysates were subjected to immunoprecipitation and Western blotting with the indicated antibodies. (B) C-terminal residual sequence from residues 658 to 878 of VP1 of IBDV. Lysine residues are displayed in boldface. (C) Screening the ubiquitin-modified K residues of VP1. 293T cells were cotransfected with HA-Ub and individual specific K-to-R mutant constructs of VP1 for 48 h, respectively. Transfected cells were lysed and analyzed by *in vivo* ubiquitination assay and Western blotting with the indicated antibodies. (D) K751R mutant of VP1 impaired its ubiquitination. 293T cells were cotransfected with HA-Ub and WT VP1, K751R mutant VP1, and K787R mutant VP1 for 48 h, respectively. Cell lysates were subjected to *in vivo* ubiquitination assay and Western blotting with the indicated antibodies. (E) The K751R mutant of VP1 abolished K63-linked ubiquitination. 293T cells were cotransfected with HA-K63 and WT VP1 or K751R mutant VP1 for 48 h. Lysates were subjected to *in vivo* ubiquitination assay and Western blotting with the indicated antibodies.

To further identify the ubiquitination site of VP1, full-length sequence of VP1 was divided into three domains based on the previously reported viral polymerase proteins (36, 37): N-terminal residues 1 to 168 (VP1-N), the central of polymerase residues 169 to 657 (VP1-D), and the C-terminal residues 658 to 878 (VP1-C). Three subdomains were cloned into a Flag tag expression plasmid to perform *in vivo* ubiquitination assays (Fig. 3A). The results shown in Fig. 3A indicated that VP1-C was efficiently modified by ubiquitin rather than VP1-N and VP1-D. To precisely identify the ubiquitination site, we examined each lysine residue within VP1-C. Figure 3B shows a schematic diagram of the 19 lysine residues, which were mutated separately to arginine (R) to generate 19 mutants and then subjected to *in vivo* ubiquitination assays. The data shown in Fig. 3C reveal that the mutation of K751R and K787R almost abolished ubiquitination of VP1 compared to the wild-type (WT) and other lysine mutants of VP1. The precise confirmation further showed that K751R but not K787R of VP1 completely lost its ubiquitin modification (Fig. 3D) and that the K751R mutant of VP1 failed to be modified by K63-linked ubiquitin (Fig. 3D and E). Taken together, the K751 residue of IBDV VP1 is essential for ubiquitination.

### The K751 ubiquitination of VP1 is essential for its polymerase activity.

**FIG 4:**
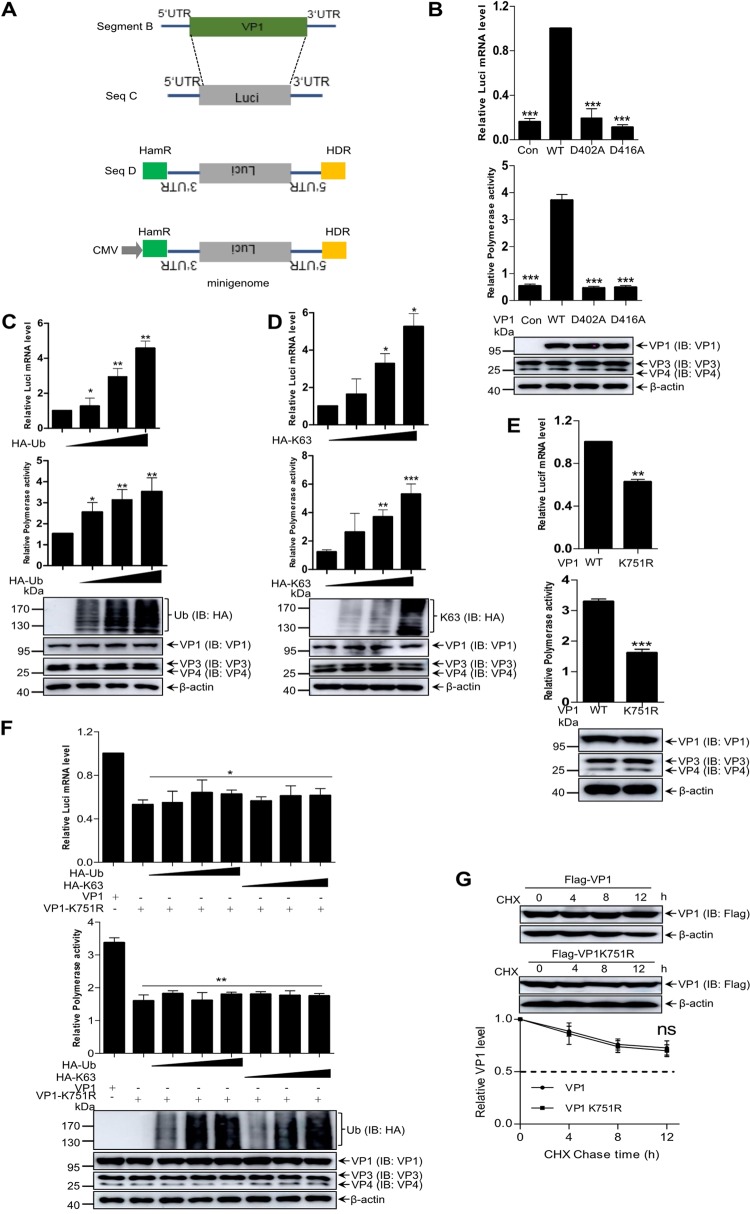
The K751 ubiquitination of VP1 is essential for its polymerase activity. (A) Schematic diagram of the minigenome reporter system. The ORF of VP1 gene between the 5′ and 3′ UTRs of IBDV genomic segment B was replaced by the ORF of the firefly luciferase gene. The resulting cassette was subcloned into vector pCDNA3.0 by an antisense orientation between HamR and HDR. (B) The minigenome reporter system could work efficiently. Western blotting, RT-qPCR, and polymerase activity assays were performed using 293T cells expressing pCDNA3.0-A, together with a minigenome reporter system and pRL-TK in the absence of VP1 (Con) or presence of VP1 (WT), D402A, or D416A mutant VP1. (C and D) Wild-type ubiquitin (C) and K63-linked ubiquitins (D) promoted VP1 polymerase activity. Polymerase activity assays were performed using 293T cells expressing segment A and segment B, along with minigenome and pRL-TK, as well as increasing amounts of HA-Ub and HA-K63. Protein expression and luciferase mRNA were analyzed by Western blotting and RT-qPCR. (E) Ubiquitin-deficient VP1 showed a significant reduction in polymerase activity. Polymerase activity and luciferase mRNA levels were assessed in 293T cells expressing IBDV K751R mutant (K751R) and WT segment B (WT), together with minigenome and pRL-TK. (F) The polymerase activity and luciferase mRNA level of the K751R mutant VP1 was not improved with an increasing expression of WT ubiquitin and K63-linked ubiquitin. Polymerase activity assays were performed using 293T cells expressing pCDNA3.0-A, pCDNA3.0-B, or K751R mutant, along with minigenome and pRL-TK, as well as increasing doses of HA-Ub or HA-K63. (G) Stability difference between K751R mutant and WT VP1. CHX assays were performed to estimate the degradation half-life of WT VP1 and K751R mutant VP1. Data are presented as the means ± the SD of three independent experiments. ns, *P* > 0.05; *, *P* < 0.05; **, *P* < 0.01; ***, *P* < 0.001.

Viral protein VP1 is involved in genome replication, gene expression, and virion maturation of IBDV. Whether ubiquitination is involved in VP1 polymerase activity was unknown. To assess the effects of ubiquitination on VP1 polymerase activity, similar to the method of IBDV genomic segment A as stated previously (38–40), we developed a minigenome report system based on genomic segment B according to the model of Fig. 4A, and both hammer head ribozyme (HamR) and hepatitis delta virus ribozyme (HDR) were used to generate minigenome report vector by inserting the luciferase gene into antisense sequence of the viral 3′ untranslated region (3′ UTR) and the 5′ UTR. Polymerase activity assays were performed, along with Western blotting and quantitative reverse transcription-PCR (RT-qPCR) assays, in the presence of WT VP1, aspartic acid 402 mutated to alanine (D402A), and aspartic acid 416 mutated to alanine (D416A) mutant VP1 (reported polymerase activity dead VP1 previously) or in the absence of VP1 (33). We found that this minigenome report system could work efficiently with low background value (Fig. 4B).

Next, we estimated the effect of VP1 ubiquitination on polymerase activity. Data shown in Fig. 4C and D show that both K63-linked and WT ubiquitin increased the polymerase activity of VP1 in a dose-dependent manner and did not change the stability of viral protein VP1, indicating that the polymerase activity of VP1 was regulated by ubiquitination. To validate whether ubiquitination at K751 of VP1 is related to its polymerase activity, we constructed the K751R mutant of VP1. We demonstrated that the K751R mutation of VP1 displayed a significantly reduced polymerase activity in comparison to that of the WT VP1, and the polymerase activity of VP1 K751R mutant could not recover with increasing doses of WT ubiquitin and K63-linked ubiquitin (Fig. 4E and F). In addition, Fig. 4 also exhibited a dynamic profile of luciferase mRNA corresponding to an alteration in polymerase activity. However, cycloheximide (CHX; a eukaryotic protein synthesis inhibitor) assays revealed that the K751 mutation did not change the stability of VP1protein (Fig. 4G). Taken together, our data demonstrated that the K751 ubiquitination in VP1 C terminus is critical for its polymerase activity.

### The K751 ubiquitination of VP1 is independent of VP1 interaction with VP3 and eIF4AII.

**FIG 5:**
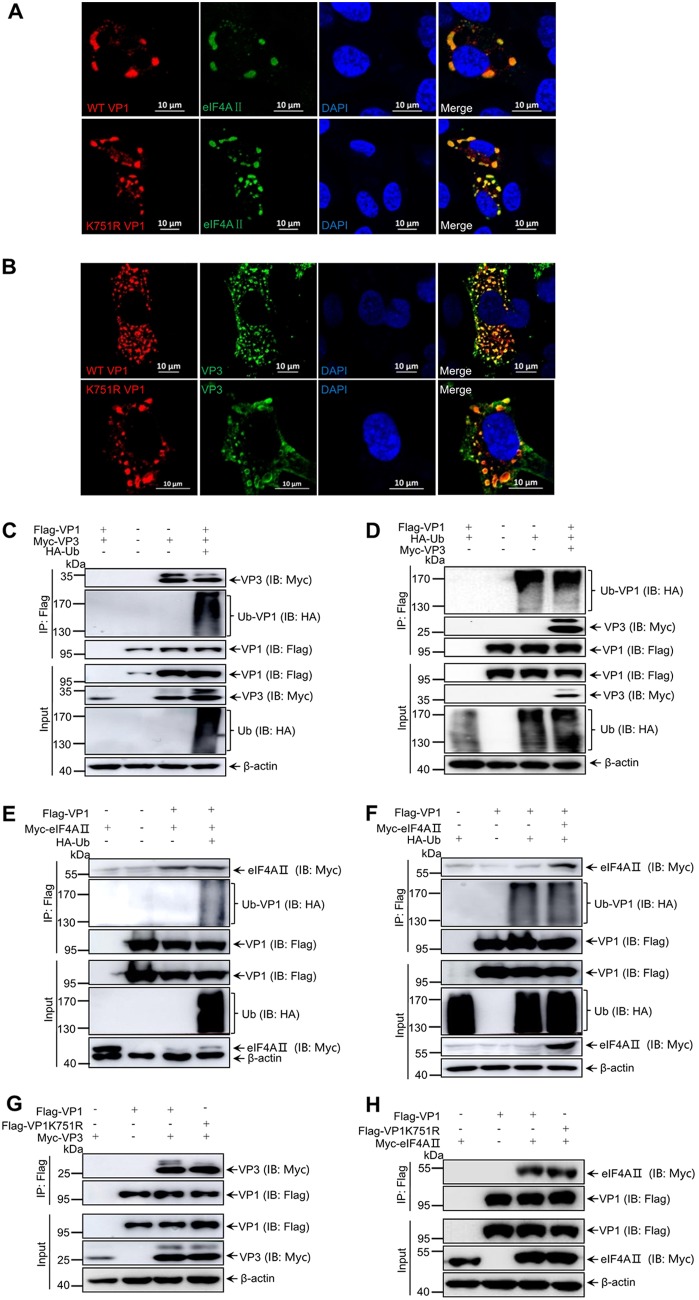
K751 ubiquitination of VP1 did not disturb VP1 interaction with VP3 and eIF4AII. (A) The K751R mutant VP1 was colocalized with eIF4AII. DF-1 cells transfected with Myc-eIF4AII for 18 h were infected with K751R mutant and WT IBDV for 12 h. Immunofluorescence analysis was performed with anti-VP1 MAb and anti-Myc pAb as the primary antibodies and FITC-labeled goat anti-rabbit and Alexa Fluor 546-conjugated anti-mouse antibodies as the secondary antibodies. (B) K751R mutant VP1 was colocalized with VP3. DF-1 cells were infected with K751R mutant and WT IBDV for 12 h. Immunofluorescence analysis was performed with anti-VP1 pAb and anti-VP3 MAb as the primary antibodies and Alexa Fluor 546-conjugated anti-mouse and FITC-labeled goat anti-rabbit antibodies as the secondary antibodies. DAPI staining revealed nuclei. Scale bars, 10 μm. (C) Ubiquitination of VP1 did not affect the formation of the VP1-VP3 complex. Fresh 293T cells were transfected by the indicated plasmids for 48 h. The lysates were subjected to immunoprecipitation with Flag beads, and Western blotting was performed with the indicated antibodies. (D) Ubiquitination of VP1 was not affected by VP3. 293T cells were transfected with indicated plasmids for 48 h. The lysates were subjected to immunoprecipitation with Flag beads, and Western blotting was performed with the indicated antibodies. (E) Ubiquitination of VP1 did not alter the interaction of VP1 with eIF4AII. The lysates were subjected to immunoprecipitation with Flag beads, and Western blotting was performed with the indicated antibodies. (F) Ubiquitination of VP1 was not affected by eIF4AII. 293T cells were transfected with the indicated plasmids for 48 h. The lysates were subjected to immunoprecipitation with Flag beads, and Western blotting was performed with the indicated antibodies. Cell lysates were immunoprecipitated with anti-Flag resin and immunoblotted with the indicated antibodies. (G and H) The K751R mutant VP1 still interacted with VP3 (G) and eIF4AII (H). Lysates from 293T cells cotransfected with Flag-VP1 or Flag-K751R VP1 and Myc-VP3 or Myc-eIF4AII for 48 h were subjected to immunoprecipitation and Western blotting with the indicated antibodies.

**FIG 6:**
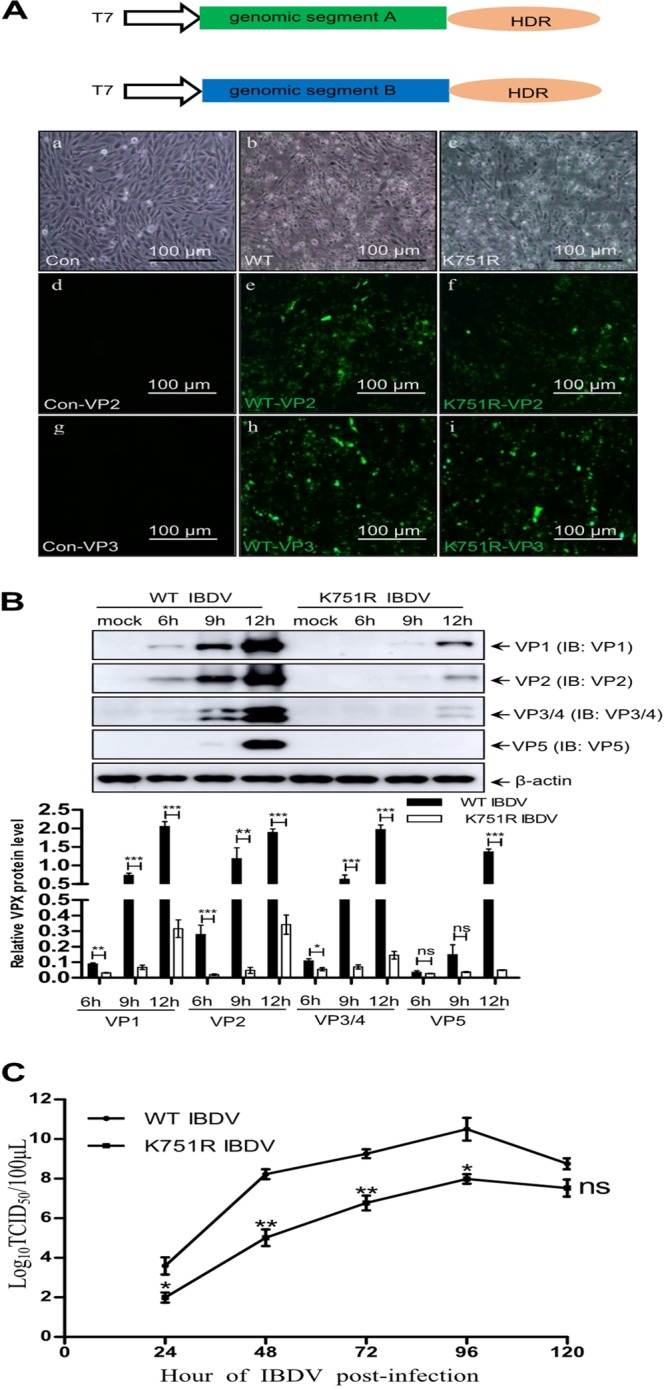
K751 ubiquitination of VP1 facilitates IBDV replication. (A) IBDV with the K751R mutation of VP1 was successfully rescued by IBDV CT stain rescue system using a T7 promoter system combined with the HDR sequence. CPE and IFAs of IBDV were observed. (a, d, and g) Mock-infected cells; (b, e, and h) WT IBDV; (c, f, and i) rescued K751 mutant IBDV. Scale bar, 100 μm. (B) Western blotting of viral proteins from K751R and WT IBDV. DF-1 cells were infected with K751R IBDV or WT IBDV at 1 MOI and cultured for the indicated times. Cell lysates were subjected to Western blotting with the indicated viral protein antibodies. Data are presented as the means ± the SD of three independent experiments. ns, *P* > 0.05; *, *P* < 0.05; **, *P* < 0.01; ***, *P* < 0.001. β-Actin was used as a loading control. (C) One-step growth curves of WT and mutant IBDV. DF-1 cells were infected with WT and K751R mutant IBDV at 1 MOI, respectively. The cells were harvested at the indicated time points, and the TCID_50_ was calculated as described in Materials and Methods. Data are presented as the means ± the SD of three independent experiments. ns, *P* > 0.05; *, *P* < 0.05; **, *P* < 0.01.

Viral protein VP1 of IBDV was previously reported to interact with viral protein VP3 and eIF4AII (41, 42). To determine whether VP1 interaction with VP3 or eIF4AII affects K751 ubiquitination, the recombinant vectors HA-Ub, Myc-VP3, or Myc-eIF4AII and Flag-VP1 were cotransfected into 293T cells. Overlapping assays showed that the WT VP1 and the K751R mutation VP1 efficiently colocalized with VP3 or eIF4AII in DF-1 cells infected with rescued K751R VP1 mutant IBDV (Fig. 5A and B; Fig. 6). Further coimmunoprecipitation (Co-IP) assays exhibited that VP1 ubiquitination was not disturbed by VP1’s interaction with VP3 or eIF4AII in the cotransfected cells with ubiquitin overexpression (Fig. 5C to F). Furthermore, VP1 protein with the K751R mutation still interacted with VP3 and eIF4AII (Fig. 5G and H). Thus, our data demonstrated that the K751 ubiquitination is independent of VP1 binding to VP3 or eIF4AII and did not affect VP1-VP3 and VP1-eIF4AII complex formation.

### The K751 ubiquitination of VP1 facilitates IBDV replication.

To investigate the function of VP1 ubiquitination in IBDV replication, we constructed and rescued IBDV with the K751R mutation of VP1 (K751R virus) using reverse genetics. T7 RNA polymerase promoter rescue plasmids T7-A and T7-B or T7-B-K751R mutant plasmid were cotransfected into BSRT7 cells (stably expressing T7 RNA polymerase) for 72 h to subsequently infect fresh DF-1 cells (43). Figure 6A showed that the cytopathic effect (CPE) and strong VP2 and VP3 signal determined by immunofluorescence assays (IFAs) was observed in infected DF-1 cell monolayers. To further identify characteristics of rescued WT and K751 viruses, we analyzed their virus replication abilities. In a Western blot assay, the expression levels of viral proteins VP1, VP2, VP3, VP4, and VP5 were significantly decreased in K751R virus-infected cells at an early stage of infection compared to WT IBDV-infected cells (Fig. 6B). Consistently, detection of virus titer also showed that the 50% tissue culture infective dose (TCID_50_) of the K751R virus was significantly lower than that of WT IBDV during replication, indicating that the K751R mutation of VP1 weakened the replication ability of IBDV (Fig. 6C). Taken together, these data indicate that the K751R mutation of VP1 did not inactivate IBDV replication and that K751 ubiquitination of VP1 improved IBDV replication.

## DISCUSSION

VP1, the RdRp of IBDV, is responsible for viral genome replication and transcription and is a core component of IBDV replication complex during infection (44, 45). Current knowledge of viral RdRp ubiquitination is limited to RdRp of avian influenza virus (9, 46–48) and polymerase cofactor VP35 of Ebola virus (14). However, there has been only one report involving self-guanylylation of VP1. In the present study, we provided evidence for the regulation of VP1 activity of IBDV via ubiquitination.

Ubiquitin is covalently attached to the target protein. The molecular mass shift of the target protein is ∼8 kDa once a ubiquitin molecule is attached. Moreover, different types of ubiquitin modification play different biological roles during virus infection. The E3 ubiquitin ligases TRIM22 and TRIM32 have been demonstrated to have antiviral functions by promoting K48-linked ubiquitination of the nucleocapsid protein (NP) and RNA polymerase subunit 1 (PB1), respectively (47, 48). Monoubiquitination of NP K184 was reported to improve viral polymerase activity (9). In the present study, we demonstrated that ubiquitination of VP1 could be detected in IBDV-infected cells and target tissue, but not in purified virions, which suggested that ubiquitination of VP1 did not affect the assembly of IBDV virions and was not a structural component of IBDV particles. An explanation is that the ubiquitin-linked VP1 molecule is only related to its polymerase activity and does not require the assembly of an IBDV virion. Similarly, avian influenza virus proteins NP and PB1 could be efficiently modified by ubiquitin during replication, and these ubiquitinated proteins were not incorporated into mature viral particles (47–49). Interestingly, ubiquitinated Gag protein of human immunodeficiency virus 1 could be efficiently packaged into mature particles (13, 50).

The K48-linked ubiquitin chain is commonly involved in protein degradation via proteasomes, while the K63-linked ubiquitin chain is associated with protein trafficking, folding maturation, and interaction of target proteins. In this study, VP1 was shown to be majorly modified by K63-linked ubiquitin but not by K48-linked ubiquitin. Moreover, we further observed that overexpressing K63-linked ubiquitin could significantly promote VP1 polymerase activity in a dose-dependent manner. These data demonstrate that the K63-linked ubiquitin chain plays an important role in maintaining VP1 polymerase activity of IBDV.

VP3 of IBDV and host eIF4AII have been reported to promote and inhibit VP1 polymerase activity, respectively (32, 42). In present study, K751 in the C terminus of VP1 was identified as the target site for K63-linked ubiquitin (Fig. 3); however, K751 ubiquitination of VP1 was not disturbed when VP1 interacted with VP3 or eIF4AII (Fig. 5). These data implied not only that VP1’s interactions with VP3 and eIF4AII are not regulated the K751 ubiquitination but also that VP1 polymerase activity regulated by the K751 ubiquitination was irrelevant to VP1’s interaction with VP3 and eIF4AII. Notably, in a reverse genetics experiment, IBDV could be rescued when K751 was mutated to R751, but the virus titer of the K751R mutant IBDV was significantly decreased compared to that of the WT IBDV, demonstrating that the K751R mutation of VP1 is not lethal to IBDV replication, although it attenuates the replication ability of mutant IBDV. Whether the low virus titer of the K751R mutant IBDV affects its virulence requires further investigation.

In conclusion, we identified that the IBDV polymerase protein was modified by K63-linked ubiquitin. The K751 residue at C-terminal of VP1 was the target site of K63-linked ubiquitin and sustained the polymerase activity of VP1 via ubiquitination. In addition, the K751 ubiquitination of VP1 is important to control IBDV replication. These results not only increased our understanding of ubiquitination in mediating IBDV RNA polymerase activity but also identified a novel target site for generating attenuated virus strains to control IBDV transmission.

## MATERIALS AND METHODS

### Cells and viruses.

HEK293 cells (a human embryonic kidney cell line; ATCC [American Type Culture Collection, Manassas, VA] CRL-11268) were routinely maintained in Dulbecco modified Eagle medium (DMEM; Gibco, Carlsbad, CA) supplemented with 10% fetal bovine serum (FBS) (catalog no. 1616756; Biological Industries, Israel, IL). The chicken fibroblast cell line DF-1 (ATCC; CRL-12203) was also cultured with 10% FBS (CCS30010.02; MRC, Australia). The T7 RNA polymerase stably expressing cell line, BSRT7 (kindly provided by Jingjing Cao, Shandong University, China) was cultured in DMEM containing 10% FBS (Biological Industries) and 500 μg/ml G418 (A100859; Sangon Biotech, Shanghai, China) for selection. All the cells were cultured in 37°C with 5% CO_2_. IBDV strain NB (NB virus) was isolated by the Key Laboratory of Animal Virology (Hangzhou, China). IBDV strain CT (CT virus) was generously provided by Bernard Delmas.

### Antibodies and reagents.

Rabbit polyclonal antibody (pAb) against Myc (R1208-1), GAPDH (glyceraldehyde-3-phosphate dehydrogenase; EM1101), and β-actin (EM21002) were purchased from Huaan Biological Technology (Hangzhou, China). Mouse anti-HA (H3663) and anti-Flag (F1804) MAbs were both obtained from Sigma-Aldrich (USA). Anti-Flag affinity resin (A2220) for immunoprecipitation was also purchased from Sigma-Aldrich. Rabbit pAb against ubiquitin (ab7780) were purchased from Abcam (USA). Rabbit anti-K63 (D7A11) and anti-K48 (D9D5) MAbs were obtained from Cell Signaling Technology (USA). Rabbit pAb to VP1 of IBDV, chicken pAb to VP3 of IBDV, and mouse MAb to VP1, VP2, VP3, VP4, and VP5 of IBDV were produced by our laboratory (17, 51, 52). MG132 (S2619) was purchased from Selleckchem (USA). Cycloheximide (CHX) was obtained from Medchemexpress (HY-12320). *N*-ethylmaleimide (NEM), a deubiquitination inhibitor, was obtained from Sigma-Aldrich (E3876). Cell NP-40 lysis buffer (50 mM Tris [pH 7.4], 150 mM NaCl, 1% NP-40) was purchased from Beyotime (P0013F; Shanghai, China). Horseradish peroxidase (HRP)- or fluorescein isothiocyanate (FITC)-labeled goat anti-mouse and anti-rabbit IgG were purchased from KPL (Milford, MA). FITC-labeled goat anti-chicken antibodies were purchased from Abcam (ab6873). Alexa Fluor 546-conjugated anti-mouse or anti-rabbit IgG were obtained from Invitrogen (USA).

### DNA construction and transfection.

cDNA fragments encoding all the IBDV viral proteins derived from the NB virus, were amplified by PCR and subcloned separately into the vector p3*Flag-cmv-10 (E7658; Mission Collection, St. Louis, MO). Vectors encoding Myc-VP3 and Myc-eIF4AII were constructed in our laboratory (unpublished data). Plasmids HA-Ub, HA-K48, HA-K63, HA-K63R, and HA-K63R were kindly provided by Hongbin Shu (53). Several Flag-VP1 mutants were created using site-specific mutagenesis. The minigenome reporter plasmid used in the polymerase activity assays was constructed according to the model shown in Fig. 4A (38). Briefly, the full-length open reading frame (ORF) of VP1 was replaced by the full-length ORF of luciferase (Luci) derived from vector pGL3.0-basic (Promega) by PCR using a pair of primers (5′-ATGGAAGACGCCAAAAACATAAAGA-3′ and 5′-TTACACGGCGATCTTTCCGCCCTTC-3′) to produce sequence C (Seq C). Subsequently, hammerhead ribozyme (HamR; 5′-TGTTAAGCGTCTGATGAGTCCGTGAGGACGAAACTATAGGAAAGGAATTCCTATAGTC-3′) and hepatitis delta virus ribozyme (HDR; 5′-GGGTCGGCATGGCATCTCCACCTCCTCGCGGTCCGACCTGGGCATCCGAAGGAGGACGCACGTCCACTCGGATGGCTAAGGGAGGGCG-3′) sequences were synthesized and ligated to the 5′ and 3′ ends of the reverse complementary sequence of sequence C to produce sequence D (Seq D). Next, Seq D was inserted into the vector pCDNA3.0 via EcoRI and XhoI to generate the minigenome reporter vector using a pair of primers (5′-GCGAATTCTGTTAAGCGTCTGATGAGTCCGTGA-3′ and 5′-GCCTCGAGCGCCCTCCCTTAGCCATCC GAGTGG-3′). Finally, full-length genomic segments A and B of IBDV were inserted into plasmid pCDNA3.0 to produce pCDNA3.0-A and pCDNA3.0-B, respectively, to analyze the polymerase activity. All plasmids were transfected into cells using ExFect transfection reagent (T101-01/02; Vazyme Biotechnology, Nanjing, China) according to the manufacturer’s instructions.

### Polymerase activity assays.

The luciferase reporter gene is flanked between *cis*-acting replication elements that could be recognized by viral polymerase protein. The expression level of the luciferase gene indicated the polymerase activity. 293T cells were transfected with minigenome, pCDNA3.0-A, and pCDNA3.0-B (or K751R mutant), as well as increasing ubiquitin expression plasmids. pRL-TK was used as an internal control producing *Renilla* luciferase for normalizing cell viability and transfection efficiency. At 36 h posttransfection, the transfected cells were harvested, and the luciferase activity was measured using a dual luciferase reporter kit (DL101-01; Vazyme Biotechnology, Nanjing, China). All experiments were performed in triplicate.

### RT-qPCR.

Fresh 293T cells were transfected with indicated plasmids for 36 h. Total cellular RNA was isolated by the TRIzol reagent (Invitrogen) according to the manufacturer’s instructions. DNase I (M0303; NEB) was used to remove DNA. Reverse transcription of 1 μg of total RNA was performed using a RevertAid RT reverse transcription kit (Thermo Fisher, K1691). The relative abundance of transcripts was assayed using the ChamQ Universal SYBR qPCR master mix (Q711-02/03; Vazyme Biotechnology) and the LightCycler 96 sequence detector system (Roche). The primers 5′-CACGAAATTGCTTCTGGTGGCGCTC-3′ and 5′-CGGTTTATCATCCCCCTCGGGTGTA-3′ were used to detect luciferase mRNA, and the primers 5′-GTCAGCCGCATCTTCTTTTG-3′ and 5′-GCGCCCAATACGACCAAATC-3′ were used to detect *gapdh* mRNA.

### *In vivo* ubiquitin assays, Co-IP, and Western blotting.

For coimmunoprecipitation (Co-IP), HEK293T cells were transfected with the indicated plasmids for 48 h. Cells were lysed in NP-40 buffer (50 mM Tris [pH 7.4], 150 mM NaCl, 1% NP-40) containing a protease inhibitor cocktail. Clarified buffer lysates were precleared and immunoprecipitated using anti-Flag beads. The beads were washed three times with NP-40 buffer and then boiled in protein loading buffer before being subjected to Western blotting.

For the *in vivo* ubiquitination assays, HEK293T cells were transfected with HA-Ub or a plasmid encoding the indicated Flag-tagged viral protein for 48 h, and DF-1 cells were infected with IBDV at the indicated time points. The cells were harvested in NP-40 buffer containing protease inhibitors and 5 mM NEM and then centrifuged, and the insoluble fraction was removed. Immunoprecipitation and Western blot analyses were then performed.

For Western blotting, cell lysates in protein loading buffer were subjected to SDS-PAGE and transferred to a nitrocellulose membrane. After being blocked with 5% skimmed milk, the membranes were incubated with the indicated primary antibodies at room temperature for 1 to 3 h at 4°C overnight. After three to five washes with phosphate-buffered saline (PBS) containing 0.1% Tween 20 (PBST), the membranes were incubated with HRP-labeled secondary antibody at room temperature for 1 to 3 h. The immunoreactive protein bands were then visualized using enhanced chemiluminescence and imaged using AI600 Images (GE Healthcare).

### Immunofluorescence assay and confocal microscopy.

To observe the colocalization of eIF4AII and VP1 proteins, DF-1 cells were transfected with the vector encoding Myc-eIF4AII for 12 h and then infected with the indicated virus for 18 h. The cells were then fixed using 4% paraformaldehyde for 10 min and permeabilized with 0.2% Triton X-100 for 5 min at room temperature, based on previous research (45). The fixed cells were incubated with rabbit anti-Myc antibodies, as well as anti-VP1 MAb or pAb, at 37°C for 1 h. After three washes with PBST, the cells were incubated with FITC-labeled goat anti-rabbit and Alexa Fluor 546-labeled anti-mouse IgG at 37°C for 1 h; the nuclei were then stained with 4’,6’-diamidino-2-phenylindole (DAPI). The cells were then observed under a Nikon laser confocal microscope (Japan).

### CHX chase assays.

To study the half-life of VP1 and K751R VP1, CHX chase experiments were performed. Briefly, 293T cells were cultured in 24-well plates. Vectors encoding WT Flag-VP1 and K751R Flag-VP1 were individually transfected into cells. At 24 h posttransfection, the cells were treated with 10 μg/ml CHX dissolved in dimethyl sulfoxide. The cells were lysed at the indicated time points and subjected to immunoblotting. The protein levels were determined quantitatively using ImageJ software.

### IBDV purification.

Purification of IBDV virions was performed by sucrose density gradient centrifugation, as previously described (46). Briefly, DF-1 cells were infected with IBDV at 1 MOI and harvested when the CPE was displayed completely. After freeze-thawing three times and removing cell debris by low-speed centrifugation, the virus was pelleted by ultracentrifugation at 30,000 × *g* for 2 h at 4°C and resuspended in PBS.

### Generating mutant IBDV.

The IBDV CT strain Reverse Genetics Operations was kindly by Bernard Delmas (26). The K751 residual of genomic segment B of the CT strain virus was mutated to R. The WT T7-B and mutant K751R T7-B were individually cotransfected with T7-A into BSRT7 cells for 72 h, with T7-B alone as a negative control. After freeze-thawing the BSRT7 cells and centrifugation to remove the insoluble fractions, the supernatant was transferred into fresh DF-1 cells for 1 to 3 h, and these cells were added to fresh DMEM containing 2% FBS for continuous culture for 48 h.

### Virus growth curve.

DF-1 cells were infected with WT IBDV and K751R mutant IBDV at 1 MOI, respectively, and collected at the indicated time points. The samples were freeze-thawed three times and then centrifuged to collect the supernatant fraction. The TCID_50_ was calculated to draw the viral growth curve.

### Statistical analyses.

Statistical differences between experiments were validated by a Student *t* test (*, *P* < 0.05; **, *P* < 0.01; ***, *P* < 0.001; ns, *P* > 0.05). Each experiment was repeated three times. The results of various analyses, including CHX assays, dual-luciferase reporter assays, protein level assessments, and virus titers, are presented as means ± the standard deviations (SD).
